# From prescription to trend: the misuse of Ozempic in the age of social media

**DOI:** 10.1097/MS9.0000000000003801

**Published:** 2025-08-29

**Authors:** Hassan E Muhammad, Asad Ali Ahmed Cheema

**Affiliations:** a Department of Medicine, Gujranwala Medical College, Gujranwala, Pakistan; b Department of Medicine, International School of Medicine, International University of Kyrgyzstan, Bishkek, Kyrgyzstan

*To the Editor,*

The rising off-label use of Ozempic, a glucagon-like peptide 1 (GLP-1) receptor agonist, by healthy individuals for aesthetic weight loss, represents a troubling intersection of pharmacology and social media influence. Semaglutide is FDA-approved under the brand names Ozempic for the treatment of type 2 diabetes and Wegovy for the management of obesity. It is increasingly being used without medical indication, often influenced by social media trends and celebrity endorsements^[1]^. In accordance with the TITAN 2025 guideline, this manuscript ensures transparency in the reporting of AI involvement^[2]^.

This trend underscores a growing normalization of prescription drugs for non-therapeutic, aesthetic purposes. However, GLP-1 receptor agonists are clinically effective for obesity and metabolic syndromes in indicated patients^[3]^, their inappropriate use by healthy individuals without supervision poses serious risks. Reported adverse effects include nausea, vomiting, dehydration, headaches, and more severe outcomes such as acute pancreatitis, gallstones, and renal impairment^[4,5]^. A recent pharmacoepidemiological review by Echeverry-Guerrero et al. underscores these concerns, noting that the misuse of GLP-1 analogs may expose individuals without metabolic disease to adverse effects for which they lack protective physiological buffers^[5]^.

Of particular concern is how social media rebrands this metabolic drug as a quick-fix lifestyle enhancer. This reframing not only distorts public understanding but may contribute to overprescription, unnecessary exposure to side effects, and medication shortages. As shown in multiple studies, misleading portrayals of semaglutide have led to inequitable access, where those with financial means obtain the drug off-label. At the same time, patients with diabetes or obesity face barriers due to limited supply.^[5–7]^ The resulting strain on the healthcare system highlights broader ethical and economic implications, especially in low-resource settings where the off-label diversion of essential medication compromises therapeutic availability for those with genuine clinical needs^[5]^.

Addressing this misuse requires a multidisciplinary response. Healthcare professionals must remain vigilant and advocate against the misappropriation of pharmacologic agents driven by aesthetic or social media trends. Regulatory bodies and digital platforms share a responsibility to curb the spread of misleading content and promote medically accurate information. Most importantly, healthcare-led public awareness campaigns on digital platforms are urgently needed to counteract this phenomenon before the misuse of aesthetic pharmacology becomes normalized. As emphasized by Echeverry-Guerrero et al., implementing rational drug use (RDU) strategies and regulatory safeguards is essential to protect public health and ensure ethical evidence-based prescribing^[5]^.

## Data Availability

Not applicable – No new data were generated or analyzed in this letter to the editor.

## Peer and provenance statement

This article was not commissioned and underwent external peer review.

## Assistance with study

The authors gratefully acknowledge the guidance and mentorship provided by the Research Council of Pakistan (RCOP).
